# Stiffness analysis and structural optimization design of an air spring for ships

**DOI:** 10.1038/s41598-024-62581-3

**Published:** 2024-06-25

**Authors:** Yuqiang Cheng, Hua Gao, Jianguo Ma, Changgeng Shuai

**Affiliations:** 1 Institute of Noise and Vibration, Naval University of Engineering, Wuhan, 430033 China; 2 State Key Lab of Ship Vibration and Noise, Wuhan, 430033 China

**Keywords:** Air spring, Fiber-reinforced composite, Stiffness characteristics, Strength characteristics, Optimization design, Precise transfer matrix, Mechanical engineering, Materials science, Theory and computation

## Abstract

An air spring (AS) for ships must have the structural strength of its bellows enhanced considerably to ensure its reliability under high internal pressure and strong impact. In this case, the stiffness of the bellows gradually dominates the overall stiffness of the AS. Nevertheless, the parameterization calculation of stiffness for an AS mainly focuses on its pneumatic stiffness. The bellows stiffness is normally analyzed by virtue of equivalent simplification or numeric simulation. There is not an effective parameterization calculation model for the stiffness of the bellows, making it difficult to achieve the structural optimization design of the bellows. In this paper, the shell theory was borrowed to build a mechanical model for the bellows. Subsequently, the state vector of the bellows was solved by precision integration and boundary condition. Iteration was conducted to identify the complex coupling relationship between the vector of the bellows and other parameters. On this basis, the parameterization calculation method was introduced for the stiffness of the bellows to obtain the vertical and horizontal stiffness of the AS. After that, a dual-membrane low-stiffness structure was designed to analyze the dominating factors affecting the strength and stiffness of the AS, which highlighted the way to the low-stiffness optimization design of high-strength ASs. In the end, three prototypes and one optimized prototype were tested to verify the correctness of the parameterization design model for stiffness as well as the effectiveness of the structural optimization design.

## Introduction

An AS have been applied in the vibration isolation apparatuses of vehicles and ships to effectively interrupt the vibration transfer of large equipments^1–3^. In order to fit into the ships with confined space, an AS for ships must be strictly sized to properly support the large power equipments onboard. It must be highly capable, so that it is normally subject to very high internal pressure, more than three times of that for vehicles. Additionally, the safety coefficient of an AS for ships is often required to be greater than 10 because of the harsh cabin environment. This is much higher than the requirement for an AS for vehicles, that is, three times of working pressure under extreme conditions. Therefore, a more stable single chamber structure is normally designed for an AS for ships, which greatly enhances the structure of bellows compared with an AS for vehicles^4^.

Presently, the theoretical design of mechanical properties for an AS is mainly developed for vehicles. An AS for vehicles requires a much lower strength of bellows than an AS for ships, and the structure of its bellows has a very little effect on its stiffness. In order to ensure the reliability of an AS for ships under high internal air pressure and strong external impact, the strength of its bellows is constantly enhanced, which increases the effect of bellows on the stiffness of the AS. As a fiber-reinforced rubber composite, the bellows of an AS is typically anisotropic, making it very complicated to construct its theoretical model. For this reason, AS has been developed for more than a century, but the studies on its stiffness still focus on the calculation and analysis based on thermodynamic equation. There is not any noticeable theoretical breakthrough regarding the establishment and resolution of the stiffness model for bellows^5–9^. Benjamin^10^ took the lead in exploring the functional relationship between the bearing capacity and air pressure of an AS, put forward the concept of effective area, and conducted many tests to explore it. Shimozaw, Nieto, and Quaglia further analyzed and studied the effective area of an AS, and found that the effective area is unrelated to the internal pressure of the AS, but depends much on the height where the AS is operating^11–13^. Lee^14^ solved the vertical stiffness of an AS using an pneumatic model, and analyzed how the variation of air heat transfer and effective radius affects the vertical stiffness. Li et al*.*^7,15^ presented a method for calculating the vertical stiffness of an AS, and pointed out that the effect of rubber bellows on stiffness in the process of deformation should not be ignored while solving the stiffness. However, it was very complicated to establish a theoretical model for anisotropic bellows, so that the conclusions were verified only by numerical simulation and test results.

In recent years, some scholars have begun to analyze the effect of bellows on the stiffness of an AS by constructing a mechanical model for bellows. However, most models are established by virtue of equivalent simplification, numerical simulation, or curve fitting. A parameterization design model has not been built for bellows, making it difficult to guide the optimization of structural parameters for the design of bellows. For instance, Erin et al*.*^16^ utilized the parallel connection of linear spring and damper to equivalently simulate the mechanical model for bellows with nonlinear mechanical properties. Moreover, they solved and analyzed the stiffness of an AS. Zhu et al*.* took into account the thermodynamic effect of air inside an AS and the frictional and visco-elastic effect of rubber bellows. The employed a statistical method to determine the friction model parameters of equivalent bellows, solved and analyzed the stiffness^17^. Chen et al*.*^18^ proposed a rubber bellow model formed by a fractional Kelvin-Voigt model and a smooth friction model to solve the vertical stiffness of an AS. Based on the thermodynamic equation, Qi et al*.*^19^ derived the expression for the pneumatic stiffness of an AS, and performed the curve fitting with the results of numerical simulation to obtain the stiffness of bellows. In the end, a prototype test was conducted to verify the effectiveness of the established stiffness model. Wong et al*.*^20^ used the Rebar unit in the ABAQUS software to established a finite element model for the rubber bellows to analyze the stiffness properties of the AS.

The bellows of an AS is made of fiber-reinforced rubber composite, and have the thickness much lower than its curvature radius. Essentially, the bellows can be abstracted into a structure of fiber-reinforced composite shell. Presently, scholars have extensively studied the modeling methods and analyzed the mechanical properties of the fiber-reinforced composite shell. If the research findings of the shell theory are applied in the theoretical modeling and performance analysis of the bellows of the AS, it is possible to realize the parameterized design and structural optimization of stiffness for the AS for ships. However, the studies on the mechanical properties of the fiber-reinforced composite shell mainly concentrate on the performance analysis of the fiber-reinforced composite hose^21^. For instance, Knapp^22^ derived the stiffness matrix of the hose under tensile and torsional loads, and simplified the model of the shell theory under the assumptions of small deformation and stiffness linearization, so as to analyze the mechanical properties of the composite hose and determine their values. Francesco et al*.*^23^ studied the mechanical characteristics of a flexible hose under tensile load, and analyzed the influence of external pressure on the mechanical characteristics of the flexible shell as well as the effect of different structural parameters on the tensile strength of the shell. Dong et al*.*^24^ put forward a simplified model of hose, and introduced the penetration tolerance factor analysis method into the calculation of hose mechanical properties, so as to determine the stiffness of the hose in all directions. Felippa et al*.*^25^ employed the Newton–Raphson iteration method and combined it with the geometrical nonlinear coupling deformation effect. The hose stiffness matrix was constructed and solved to analyze the fiber deformation of hose under the joint effect of tensile, bending and torsional forces.

A fiber-reinforced composite hose is structurally similar to the bellows of an AS. It is also made of inner and outer protective layers and reinforcing interlayer^26^. It is normally made into a cylindrical shell with the filament wrapping of the same angle and uniform distribution. Differently, the bellows of an AS has a structure of variable radius. Because of structure and formation process, the bellows are characterized by complex variable winding trajectory. Moreover, the influence of the pre-stress caused by the bellows of an AS under preload conditions must be taken into account while solving the stiffness of the bellows. The state vector and internal pressure of the bellows also have a complex coupling relationship with the structural parameters. All these factors have considerably contributed to the complexity of constructing and solving the mechanical model of the bellows.

Based on the shell theory, the variable winding trajectory characteristics of AS bellows and bellows pre-stress are introduced. A novel parametric stiffness model of bellows with variable winding trajectory characteristics under preload conditions is constructed using the fine transfer matrix method and iterative method. Taking into account the effect of the bellows, the parametric calculation methods of the vertical and horizontal stiffness characteristics of a ship's AS are given. Additionally, this paper analyzes the factors that influence the strength and stiffness characteristics of marine ASs. For the first time, this study clarifies the design direction for low-stiffness structures of high-strength ASs. Additionally, a novel optimization design scheme for a double-membrane low-stiffness structure is proposed. The test results indicate that the optimized design prototype reduces vertical stiffness by 27% and horizontal stiffness by 75.5% at the same rated load and effective radius.

## Parameterized design model of stiffness

The stiffness of an AS contains pneumatic stiffness and bellow stiffness. It can be divided into vertical stiffness and horizontal stiffness based on the deformation direction of the AS. The stiffness is expressed by:

1 $$\left\{ \begin{gathered} K_{Z} = K_{Z}^{p} + K_{Z}^{b} \hfill \\ K_{H} = K_{H}^{p} + K_{H}^{b} \hfill \\ \end{gathered} \right.$$

where *K*_*Z*_ and *K*_*H*_ are the vertical and horizontal stiffness, respectively; $K_{Z}^{p}$ and $K_{H}^{p}$ are the vertical and horizontal pneumatic stiffness, respectively; $K_{Z}^{b}$ and $K_{H}^{b}$ are the vertical and horizontal bellow stiffness, respectively. Presently, scholars have carried out the extensive and profound studies on the pneumatic stiffness model for an AS, and obtained the accurate equations for the parameterized calculation of pneumatic stiffness^19^. For this reason, attention is paid to how to solve the bellow stiffness of an AS in this section.

### Building a theoretical model for the bellows

**Figure 1:**
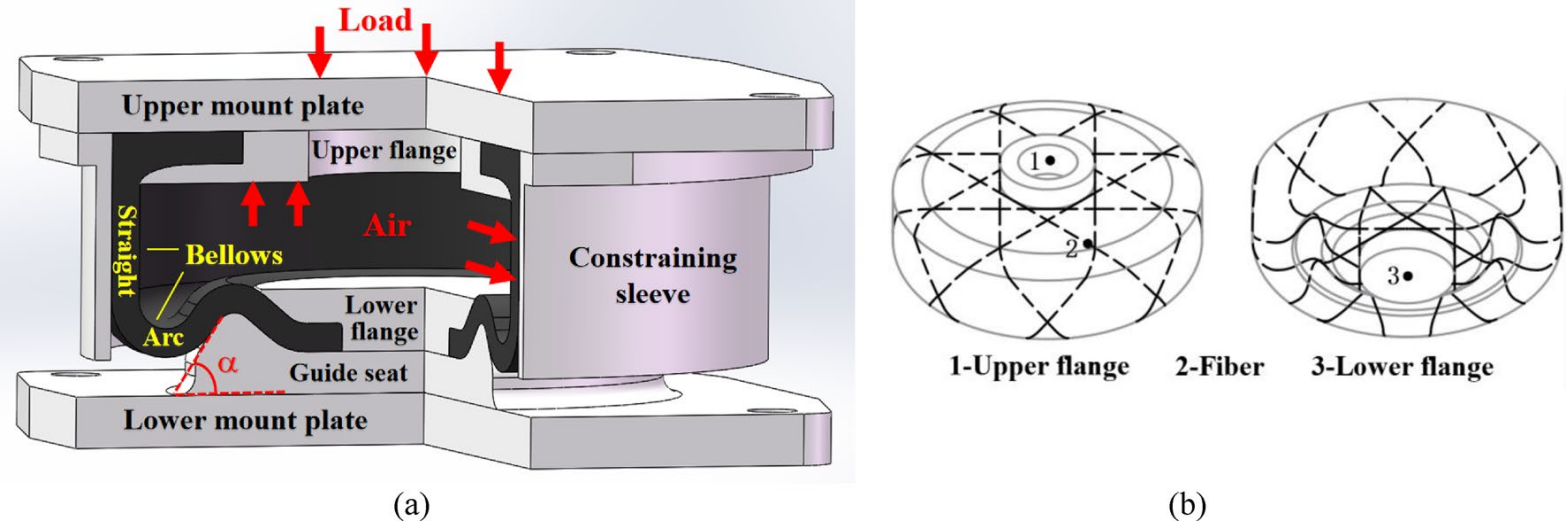
Structure of an AS for ships (**a**) AS composition; (**b**) Integrated filament winding reinforced bellows.

As shown in Fig. 1, an AS for ships normally consists of upper mount plate, upper flange, rubber bellows, lower flange, guide seat, lower mount plate, and constraining sleeve, etc. The internal fibers of the bellows adopt integrated winding method to bind the upper and lower flanges into a whole, with complex variable winding trajectory characteristics. The AS relies on the high-pressure air in itself to support the load of external equipment. When the bellows are deformed under the vibration of equipment, the straight part is pushed by the high-pressure air against the constraining sleeve, while the arc part curls over along the guide seat. As a result, the bellows stiffness mainly depends on the mechanical state of the arc part in the process of deformation.

**Figure 2:**
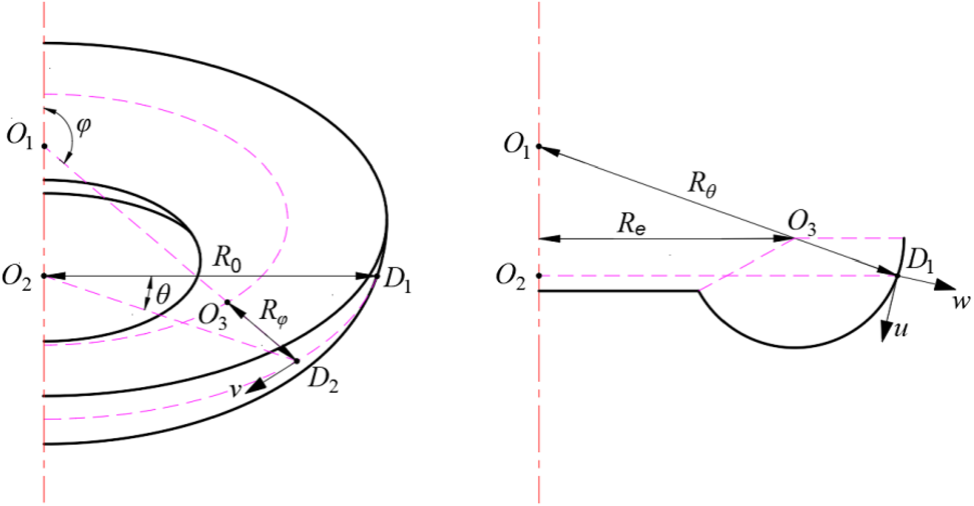
Structure diagram of the rotary shell.

As illustrated in Fig. 2, the arc part of the bellows is simplified into a rotary shell. Any point on the rotary shell is denoted by the surface coordinates (*φ*, *θ*), where *φ* stands for the coordinate in the latitudinal direction and *θ* represents the coordinate in the longitudinal direction. The principal curvature radius is *R*_*φ*_ and *R*_*θ*_, respectively. The curvature radius of the latitudinal surface is represented by *R*_*0*_. Based on the geometrical structural relationship, it is obtained that:

2 $$\left\{ \begin{gathered} R_{\theta } = R_{\varphi } + R_{e} /\sin \varphi \begin{array}{*{20}c} {} & {} \\ \end{array} (\varphi \le \pi ) \hfill \\ R_{\theta } = - (R_{\varphi } + R_{e} /\sin \varphi )\begin{array}{*{20}c} {} \\ \end{array} (\pi < \varphi ) \hfill \\ R_{0} = R_{\theta } \sin \theta \hfill \\ \end{gathered} \right.$$

An AS normally functions under the joint effect of internal pressure *P* and external load *F*. The equilibrium equation of the bellows must take into account the pre-stress in the bellows when the AS is under preload conditions. After analyzing the variation of stress and strain with the pre-stress in the equilibrium equation, third-order minute and second-order disturbance are ignored to derive the equilibrium equation of the bellows based on the Flügge theory as follows^27^:

3 $$\left\{ \begin{gathered} \frac{{\partial \left( {R_{0} N_{\varphi } } \right)}}{\partial \varphi } + R_{\varphi } \frac{{\partial N_{\theta \varphi } }}{\partial \theta } - R_{\varphi } \cos \varphi N_{\theta } + R_{0} Q_{\varphi } - PR_{0} \left( {\frac{\partial \omega }{{\partial \varphi }}} \right) \hfill \\ + N_{{\varphi_{0} }} \frac{{R_{0} }}{{R_{\varphi } }}\left( {\frac{{\partial^{2} u}}{{\partial \varphi^{2} }} + \frac{\partial \omega }{{\partial \varphi }}} \right) + \frac{{N_{{\theta_{0} }} }}{{R_{0} }}\frac{{\partial^{2} u}}{{\partial \theta^{2} }} = 0 \hfill \\ \frac{{\partial \left( {R_{0} N_{\varphi \theta } } \right)}}{\partial \varphi } + \frac{{R_{\varphi } }}{{R_{0} }}\left( {\frac{\partial u}{{\partial \theta }}\cos \varphi + \frac{{\partial^{2} v}}{{\partial \theta^{2} }} + \frac{\partial \omega }{{\partial \theta }}\sin \varphi } \right)N_{{\theta_{0} }} + R_{\varphi } \frac{{\partial N_{\theta } }}{\partial \theta } \hfill \\ + R_{\varphi } \cos \varphi N_{\theta \varphi } + N_{{\varphi_{0} }} R_{0} \frac{{\partial^{2} v}}{{\partial \theta^{2} }} + R_{\varphi } \sin \varphi Q_{\theta } = 0 \hfill \\ \frac{{\partial \left( {R_{0} Q_{\varphi } } \right)}}{\partial \varphi } - N_{{\theta_{0} }} \frac{{R_{\varphi } }}{{R_{0} }}\sin \varphi \left( {u\cos \varphi + 2\frac{\partial v}{{\partial \theta }} + w\sin \varphi - \frac{{\partial^{2} w}}{{\partial \theta^{2} }}} \right) \hfill \\ + R_{\varphi } \frac{{\partial Q_{\theta } }}{\partial \theta } - R_{\varphi } R_{0} \left( {\frac{{N_{\varphi } }}{{R_{\varphi } }} + \frac{{N_{\theta } }}{{R_{\theta } }}} \right) + R_{\varphi } P\left( {\frac{\partial u}{{\partial \varphi }} + \frac{\partial v}{{\partial \theta }} + w} \right) + R_{0} N_{{\varphi_{0} }} \left( {\pi \frac{{\partial^{2} w}}{{\partial^{2} \varphi }}} \right) = 0 \hfill \\ Q_{\varphi } = \frac{1}{{R_{\varphi } R_{0} }}\left( {\frac{{\partial \left( {R_{0} M_{\varphi } } \right)}}{\partial \varphi } + R_{\varphi } \frac{{\partial M_{\theta \varphi } }}{\partial \theta } - R_{\varphi } \cos \varphi M_{\theta } } \right) \hfill \\ Q_{\theta } = \frac{1}{{R_{\varphi } R_{0} }}\left( {\frac{{\partial \left( {R_{0} M_{\varphi \theta } } \right)}}{\partial \varphi } + R_{\varphi } \frac{{\partial M_{\theta } }}{\partial \theta } + R_{\varphi } \cos \varphi M_{\theta \varphi } } \right) \hfill \\ \end{gathered} \right.$$

where *N*_*φ*_, *N*_*θ*_ and *N*_*φθ*_ are the internal force of middle plane in the shell, respectively; *M*_*φ*_, *M*_*θ*_ and *M*_*φθ*_ are the bending moment of middle plane in the shell, respectively; *u*, *v*, and *w* are the displacement of middle plane in the shell, respectively; *Q*_*φ*_ and *Q*_*θ*_ are the shear force of middle plane in the shell, respectively; *S*_*φ*_ and *V*_*φ*_ represent the Kelvin-Kirchhoff internal and horizontal shear force of middle plane, respectively; *N*_*φ*0_ and *N*_*θ*0_ are the prestress of the bellows, respectively, and expressed by^28^:

4 $$\left\{ \begin{gathered} N_{\varphi } = P\left( {\frac{{R_{\varphi }^{2} sin(\varphi ) + 2R_{\varphi }^{{}} R_{e} }}{{2R_{\varphi }^{{}} \sin (\varphi ) + 2R{}_{e}}}} \right) \hfill \\ N_{\theta } = P\frac{{R_{\varphi } }}{2} \hfill \\ \end{gathered} \right.$$

### Solving a mechanical model for the bellows

The state vector *η*(*φ*) and intermediate vector of displacement *ξ*(*φ*) are introduced. Among them, *η*(*φ*) consists of displacement and internal force, while *ξ*(*φ*) is formed by displacement and displacement derivative as follows:

5 $$\left\{ \begin{gathered} {\varvec{\eta}}(\varphi ) = \begin{array}{*{20}c} {\begin{array}{*{20}c} {\begin{array}{*{20}c} {\begin{array}{*{20}c} {[u(\varphi )} & {v(\varphi )} \\ \end{array} } & {w(\varphi )} & {\kappa_{\varphi } (\varphi )} \\ \end{array} } & {N_{\varphi } (\varphi )} & {S_{\varphi } (\varphi )} \\ \end{array} } & {V_{\varphi } (\varphi )} & {M_{\varphi } (\varphi )]^{T} } \\ \end{array} \hfill \\ {\varvec{\xi}}(\varphi ) = \begin{array}{*{20}c} {\begin{array}{*{20}c} {\begin{array}{*{20}c} {[\begin{array}{*{20}c} {u(\varphi )} & {v(\varphi )} \\ \end{array} } & {w(\varphi )} & {\frac{\partial u(\varphi )}{{\partial \varphi }}} \\ \end{array} } & {\frac{\partial v(\varphi )}{{\partial \varphi }}} & {\frac{\partial w(\varphi )}{{\partial \varphi }}} \\ \end{array} } & {\frac{{\partial^{2} w(\varphi )}}{{\partial \varphi^{2} }}} & {\frac{{\partial^{2} \kappa_{\varphi } (\varphi )}}{{\partial \varphi^{2} }}]} \\ \end{array}^{T} \hfill \\ \end{gathered} \right.$$

Based on the geometrical equation^29^, the physical equation^30^ with variable winding trajectory, and the equilibrium Eq. (3), variables are offset and transformed to determine the conversion between *η*(*φ*) and *ξ*(*φ*) and the first-order differential equation as follows:

6 $${\varvec{\eta}}(\varphi ) = {\varvec{B}}(\varphi ){\varvec{\xi}}(\varphi )$$

7 $$\frac{{d{\varvec{\xi}}(\varphi )}}{d\varphi } = {\varvec{C}}(\varphi ){\varvec{\xi}}(\varphi )$$

In Eqs. (6) and (7), ***B***(*φ*) is the eighth-order conversion matrix, and ***C***(*φ*) is the eighth-order stiffness matrix. The elements of these matrices are listed in the Appendix. The bellows are partitioned into *M* nodes. The precision integration method^31^ is borrowed to determine the transfer relation of *ξ*(*φ*) between different nodes as follows:

8 $${\varvec{\xi}}(\varphi_{i + 1} ) = \exp ({\varvec{C}}(\varphi_{i} ) \cdot (\varphi_{i + 1} - \varphi_{i} )){\varvec{\xi}}(\varphi_{i} ) = {\varvec{T}}_{i}^{s} {\varvec{\xi}}(\varphi_{i} )$$

In Eq. (8), ***T***^***s***^ is the transfer matrix between the intermediate vectors of displacement. Equation (6) is substituted into Eq. (8) to determine the transfer relation of *η*(*φ*) between the nodes of the bellows as follows:

9 $${\varvec{\eta}}(\varphi_{i + 1} ) = {\varvec{B}}(\varphi_{i + 1} ){\varvec{T}}_{i}^{s} {\varvec{B}}^{ - 1} (\varphi_{i} ){\varvec{\eta}}(\varphi_{i} ) = {\varvec{T}}_{i} {\varvec{\eta}}(\varphi_{i} )$$

In Eq. (8), ***T*** is the transfer matrix between state vectors. During the service of an AS, the front end of the bellows deforms with the mount plate, and the back end of the bellows is fixed onto the guide seat. Assuming that the deformation of the bellows is *x*, the boundary condition at both ends of the bellows subject to vertical deformation is as follows:

10 $$\left\{ \begin{gathered} u\left( {\varphi_{1} } \right)\cos \varphi_{1} + w\left( {\varphi_{1} } \right)\sin \varphi_{1} = 0,\begin{array}{*{20}c} {} \\ \end{array} v\left( {\varphi_{1} } \right) = 0 \hfill \\ u\left( {\varphi_{1} } \right)\sin \varphi_{1} - w\left( {\varphi_{1} } \right)\cos \varphi_{1} = x,\begin{array}{*{20}c} {} \\ \end{array} \kappa_{\varphi } \left( {\varphi_{1} } \right) = 0 \hfill \\ u\left( {\varphi_{M} } \right) = 0,\begin{array}{*{20}c} {} \\ \end{array} v\left( {\varphi_{M} } \right) = 0,\begin{array}{*{20}c} {} \\ \end{array} w\left( {\varphi_{M} } \right) = 0,\begin{array}{*{20}c} {} \\ \end{array} \kappa_{\varphi } \left( {\varphi_{M} } \right) = 0 \hfill \\ \end{gathered} \right.$$

The boundary condition at both ends of the bellows subject to horizontal deformation is defined by:

11 $$\left\{ \begin{gathered} u\left( {\varphi_{1} } \right)\cos \varphi_{1} + w\left( {\varphi_{1} } \right)\sin \varphi_{1} = x,\begin{array}{*{20}c} {} \\ \end{array} v\left( {\varphi_{1} } \right) = 0 \hfill \\ u\left( {\varphi_{1} } \right)\sin \varphi_{1} - w\left( {\varphi_{1} } \right)\cos \varphi_{1} = 0,\begin{array}{*{20}c} {} \\ \end{array} \kappa_{\varphi } \left( {\varphi_{1} } \right) = 0 \hfill \\ u\left( {\varphi_{M} } \right) = 0,\begin{array}{*{20}c} {} \\ \end{array} v\left( {\varphi_{M} } \right) = 0,\begin{array}{*{20}c} {} \\ \end{array} w\left( {\varphi_{M} } \right) = 0,\begin{array}{*{20}c} {} \\ \end{array} \kappa_{\varphi } \left( {\varphi_{M} } \right) = 0 \hfill \\ \end{gathered} \right.$$

Based on the boundary condition of the bellows in Eqs. (10) and (11), Eq. (9) is used to solve the state vector at every node. The load analysis at the front end of the bellows obtains the following expression of the vertical force $F_{Z}^{b}$ and the horizontal force $F_{H}^{b}$ caused by the bellows at the time of deformation:

12 $$\left\{ \begin{gathered} F_{Z}^{b} = \int_{0}^{2\pi } {\left( {\left( {N_{\varphi } (\varphi_{1} ) + S_{\varphi } (\varphi_{1} )} \right) \cdot R_{\theta } (\varphi_{1} )} \right)d\theta } \hfill \\ F_{H}^{b} = \int_{0}^{2\pi } {\left( {\left( {V_{\varphi } (\varphi_{1} ) \cdot cos\theta - \left( {N_{\theta } (\varphi_{1} ) + S_{\varphi } (\varphi_{1} )} \right) \cdot \sin \theta } \right) \cdot R_{\theta } (\varphi_{1} )} \right)d\theta } \hfill \\ \end{gathered} \right.$$

### Solving the stiffness of the bellows of an AS

The state vector, internal pressure, and structural parameters are coupled when the bellows of an AS are deformed. Thus the overall deformation of an AS can be regarded as the superposition of *n* small deformations. It is assumed that:

1. In the process of small deformation, the arc part of the bellows always remain circular.
2. In the process of small deformation, the parameters *R*_*e*_, *R*_*φ*_ and *P* remain unchanged.

When the AS is in the process of the *j*th small deformation, its displacement changes from *x*_*j*−1_ to *x*_*j*_. Based on the assumptions (1) and (2), the AS’s parameters *R*_*e*_, *R*_*φ*_ and *P* at this time are expressed by^19^:

13 $$\left\{ \begin{gathered} P_{j} = (P_{a} + P_{j - 1} )V_{j - 1}^{\iota } /V_{j}^{\iota } - P_{a} \hfill \\ R_{e}^{j} { = }R_{{\text{e}}}^{j - 1} { + }A_{{\text{Re}}} (x_{j}^{{}} - x_{j - 1}^{{}} ),\begin{array}{*{20}c} {} \\ \end{array} R_{\varphi }^{j} = R_{\varphi }^{j - 1} + A_{R\varphi } (x_{j}^{{}} - x_{j - 1}^{{}} ) \hfill \\ A_{Re}^{Z} = \frac{cos\alpha }{{2(1 + \sin \alpha ) + (\pi /2 + \alpha )cos\alpha }},\begin{array}{*{20}c} {} \\ \end{array} A_{R\varphi }^{Z} = \frac{ - \cos \alpha }{{2(1 + \sin \alpha ) + (\pi /2 + \alpha )cos\alpha }} \hfill \\ A_{Re}^{H} = \frac{1 - (\pi /2 + \alpha )\cos \alpha - \sin \alpha }{{2(1 + \sin \alpha ) + (\pi /2 + \alpha )cos\alpha }},\begin{array}{*{20}c} {} \\ \end{array} A_{R\varphi }^{H} = \frac{1 + \sin \alpha }{{2(1 + \sin \alpha ) + (\pi /2 + \alpha )cos\alpha }} \hfill \\ \end{gathered} \right.$$

In Eq. (13), *P*_*j*_ and *V*_*j*_ are the internal pressure and volume of the AS with the displacement *x*_*j*_; *P*_*j−*1_ and *V*_*j−*1_ are the internal pressure and volume of the AS with the displacement *x*_*j-*1_; $A_{Re}^{Z}$ and $A_{R\varphi }^{Z}$ are the vertical geometrical deformation coefficients; $A_{Re}^{H}$ and $A_{R\varphi }^{H}$ are the horizontal geometrical deformation coefficients; *ι* is the thermodynamic polytropic coefficient, and its value is 1.4^5^.

During the deformation of the AS, Eq. (13) is first used to determine the parameters $R_{e}^{j}$, $R_{\varphi }^{j}$ and *P*_*j*_ in the process of the *j*th small deformation. Subsequently, Eq. (12) is employed to calculate the variation of the vertical or horizontal force of the bellows in the process of the *j*th small deformation. In the end, iteration is carried out to obtain the following expression for the bellow stiffness *K*^*b*^:

14 $$K^{b} = \sum\limits_{i = 1}^{n} {\Delta F_{j}^{b} } /x$$

## Test and verification of the theoretical model for stiffness

**Figure 3:**
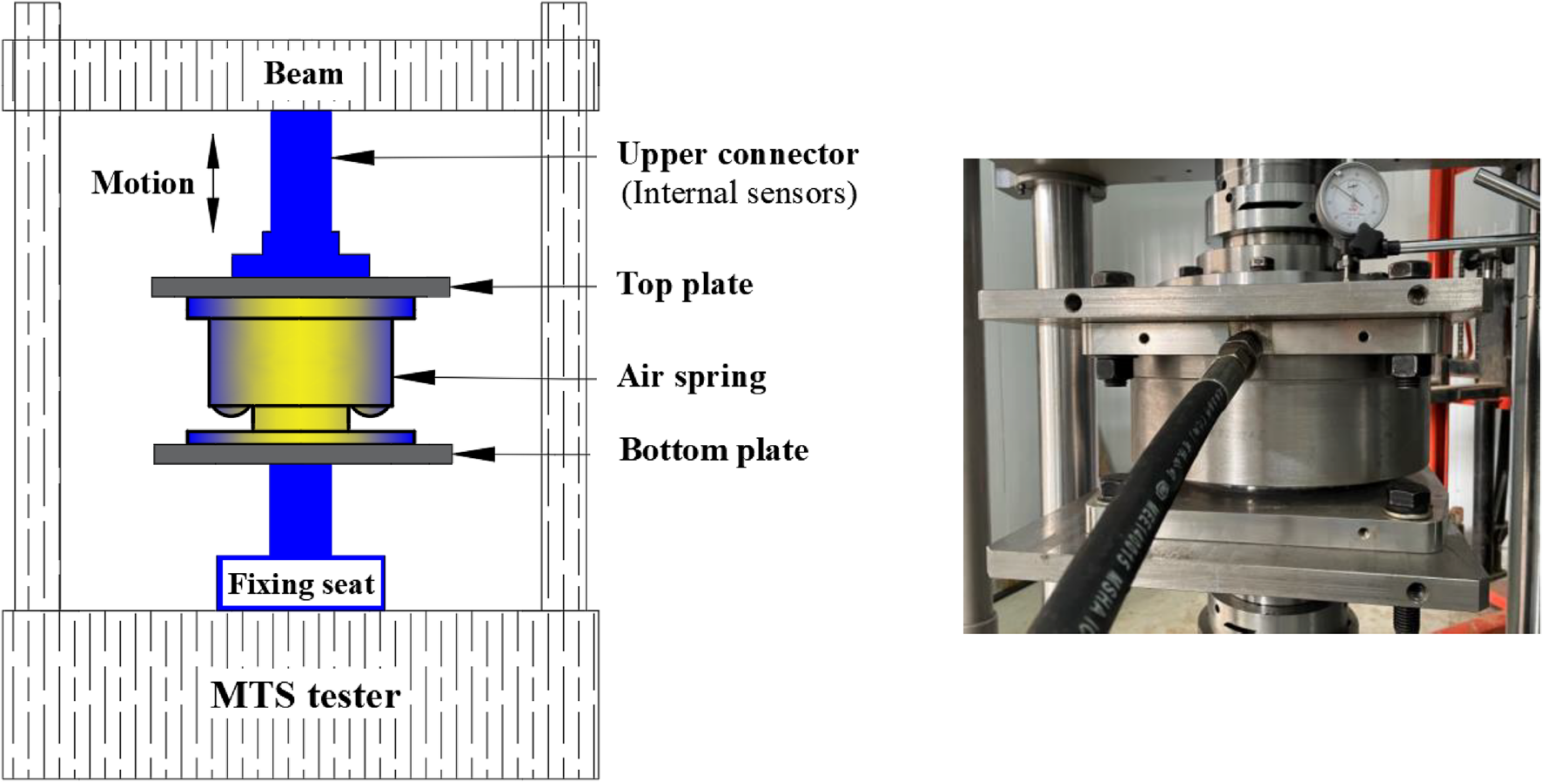
Facility and implementation of a vertical stiffness test.

**Figure 4:**
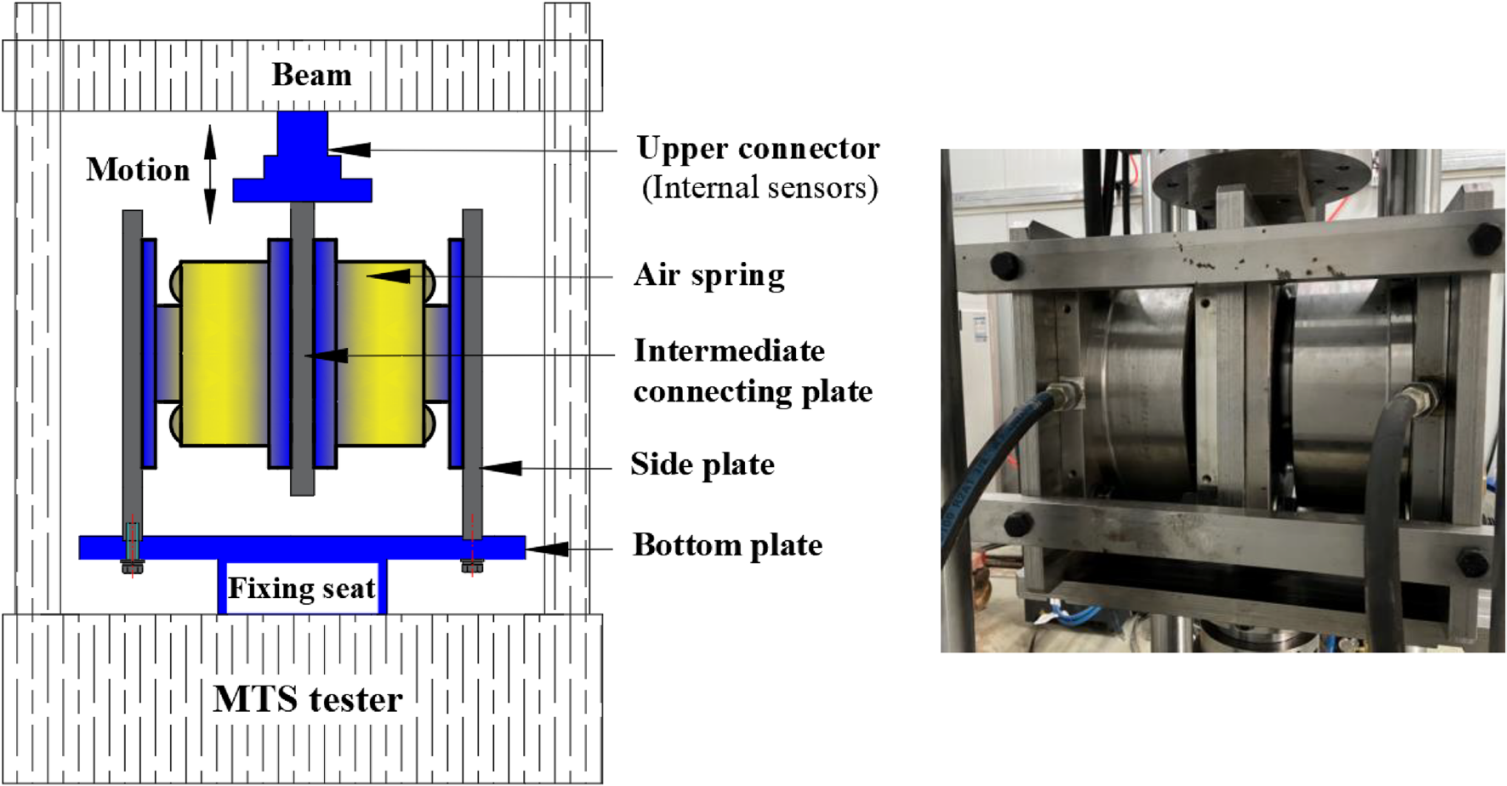
Facility and implementation of a horizontal stiffness test.

The facility and implementation of a vertical stiffness test are illustrated in Fig. 3, while the facility and implementation of a horizontal stiffness test are given in Fig. 4. During the vertical stiffness test, the upper mount plate of the AS was connected through the top plate to the upper connector of the tester. The upper mount plate reciprocated vertically with the upper grip of the tester. The lower mount plate was attached through the bottom plate to the fixing seat of the tester. During the horizontal stiffness test, two ASs were installed together. Their upper mount plates were connected through an intermediate connecting plate to the upper connector. The intermediate connecting plate reciprocated with the upper connector of the tester. The lower mount plates were connected through the side plate to the bottom plate. The bottom plate was attached to the fixing seat of the tester. During the stiffness tests, a sensor collected data and a computer output the stiffness of the AS. According to the criteria for the tests, the dynamic stiffness obtained with an excitation frequency of 3 Hz and a peak-to-peak value of 0.4 mm was taken as the vertical and horizontal stiffness of the AS in the test results, respectively.

Two 8 T, 15 T and 30 T ASs were selected for the stiffness tests. The average values were taken from test results. The structural parameters of the prototypes are given in Table 1, and the material parameters of the bellows are summarized in Table 2.

**Table 1:** Structural parameters of AS prototypes.

Parameter	8 T	15 T	30 T
Bellow radius *R*_*φ*_	26 mm	22.5 mm	32.5 mm
Effective radius *R*_*e*_	108 mm	141 mm	195 mm
Oriented angle *α*	90°	54°	60°
Thickness of filament layer *h*	6.6 mm	4.4 mm	8.8 mm
Initial winding angle *γ*	28.9°	24.8°	27.2°

**Table 2:** Material parameters of the bellows.

Parameter	Value	Parameter	Value	Parameter	Value
Elastic modulus $E$	49.7 GPa^30^	Material strength $X$	347.6 MPa^30^	Poisson’s ratio $\nu$	0.45^30^

The stiffness of the AS was tested under no load and rated load to verify whether the stiffness model was properly designed. The calculated and test results of stiffness when the AS was under no load are presented in Table 3. The internal air pressure of the AS under no load was zero, so that the calculated pneumatic stiffness was zero and the bellow stiffness is equal to the total stiffness of the AS. As shown in Table 3, the error of the calculated vertical and horizontal stiffness was less than 10%, which proved that the parameterization calculation of bellow stiffness was correct. The internal pressure of the AS under extra load was the rated working pressure. At this time, the pneumatic stiffness was not zero. The calculated and test results are detailed in Table 4. Evidently, the calculated vertical and horizontal stiffness of the AS under rated load still had an error of less than 10%, proving that the parameterized design of the model was suitable for the total stiffness of the AS.

**Table 3:** Calculated and test results of stiffness of the ASs under no load.

Description		8 T	15 T	30 T	Description	8 T	15 T	30 T
Calculated result	$K_{Z}^{p}$ (kN/mm)	0	0	0	$K_{H}^{p}$ (kN/mm)	0	0	0
	$K_{Z}^{b}$ (kN/mm)	0.79	0.98	1.23	$K_{H}^{b}$ (kN/mm)	7.05	8.27	9.49
	*K*_*Z*_ (kN/mm)	0.79	0.98	1.23	*K*_*H*_ (kN/mm)	7.05	8.27	9.49
Test result	*K*_*Z*_ (kN/mm)	0.81	1.07	1.28	*K*_*H*_ (kN/mm)	7.36	9.12	9.88
Calculation error	*K*_*Z*_	2.5%	8.4%	3.9%	*K*_*H*_	4.2%	9.3%	3.9%

**Table 4:** Calculated and test results of stiffness of the ASs under rated load.

Description		8 T	15 T	30 T	Description	8 T	15 T	30 T
Calculated result	$K_{Z}^{p}$ (kN/mm)	1.20	3.96	6.19	$K_{H}^{p}$ (kN/mm)	0.57	1.28	1.72
	$K_{Z}^{b}$ (kN/mm)	1.21	1.74	2.28	$K_{H}^{b}$ (kN/mm)	9.26	11.34	13.39
	*K*_*Z*_ (kN/mm)	2.41	5.70	8.47	*K*_*H*_ (kN/mm)	9.83	12.62	15.11
Test result	*K*_*Z*_ (kN/mm)	2.57	5.51	8.01	*K*_*H*_ (kN/mm)	10.26	13.80	16.59
Calculation error	*K*_*Z*_	6.2%	3.5%	5.7%	*K*_*H*_	4.2%	8.6%	8.9%

After further analyzing the data in Table 4, it is found that bellow stiffness is considerably improved when the bellows of the AS are structurally reinforced. Among all three types of AS, the lowest contribution of bellow stiffness to the vertical stiffness is 26.9%. In the 8 T AS, vertical bellow stiffness has been slightly higher than vertical pneumatic stiffness. It reveals that the bellow stiffness should not be ignored in the calculation of the vertical stiffness, and even plays a dominating role in the vertical stiffness. Moreover, horizontal pneumatic stiffness is much lower than horizontal bellow stiffness among all three types of AS. The bellow stiffness has been a main contributor to the horizontal stiffness. The largest contribution of the pneumatic stiffness to the total stiffness of the AS was only 11.4%, implying a very small influence of the pneumatic stiffness on the horizontal stiffness. Such influence can be even ignored in the simplified analysis of horizontal stiffness.

## Low-stiffness optimization design of high-strength bellows

### Dual-membrane low-stiffness structural design

As discussed in "Test and verification of the theoretical model for stiffness", bellow stiffness has gradually become a dominating contributor to stiffness when the bellows of an AS for ships are structurally reinforced. For this reason, effective measures must be taken to reduce the bellow stiffness, so as to properly guarantee the vibration isolation of the AS. For the reliable service of the AS, the bellow stiffness can be reduced provided that the bellows can bear the limit pressure more than ten times of its working pressure. After calculating the state vector of the bellows with Eq. (9) based on the boundary condition, the limit pressure of the bellows is calculated with the Tsai-Hill strength theory^30^.

**Figure 5:**
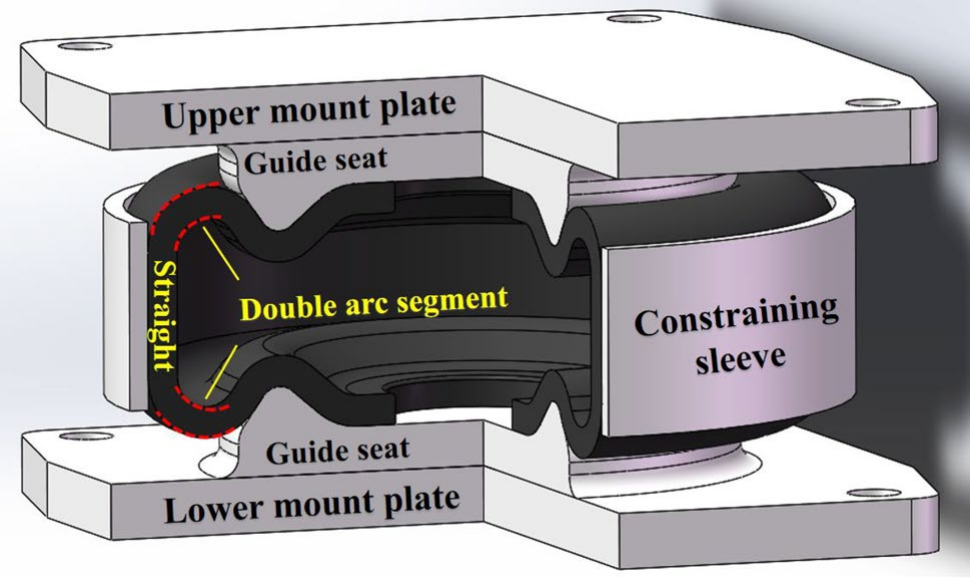
Structure of a dual-membrane low-stiffness AS.

The stiffness of high-strength bellows can be effectively lowered by optimizing the design of structural parameters or carrying out the structural optimization. Therefore, a dual-membrane low-stiffness structure is proposed in this paper. In this design, the single-membrane structure is simply changed to a dual-membrane paralleled structure. The bellow stiffness can be directly reduced by half while guaranteeing no change to the size of the AS or the theoretical model, and even having no influence on the strength of the bellows. The overall structure of the dual-membrane low-stiffness AS is illustrated in Fig. 5.

### Integrated optimization design of structural parameters

The strength and stiffness of an AS are affected by the coupling of various parameters. In the optimization design of structural parameters for an AS, it is therefore necessary to analyze the dominating factors affecting the strength and stiffness of the AS, so as to determine the focus of low-stiffness optimization design for a high-strength AS.

#### Dominating factors affecting strength and stiffness

The design parameters of an AS are classified into three categories, that is, geometrical structure parameters including effective radius *R*_*φ*_, bellow radius *R*_*e*_, and oriented angle *α*; material characteristics parameters including strength of fiber composite *X*, elastic modulus *E*, and thickness of filament layer *h*; and filament winding parameters including initial winding angle *γ*. Taking an 15 T AS as an example, a design parameter is altered to analyze how such parameter affects the strength and stiffness of the AS. In order to further simplify the analysis and highlight the dominating factor affecting the mechanical properties, the parameters of the AS prototype are normalized, and the normalized parameters are expressed by:

15 $$\left\{ \begin{gathered} R^{\prime}_{\varphi } = R_{\varphi } /R_{\varphi }^{0} ,\begin{array}{*{20}c} {} \\ \end{array} R^{\prime}_{e} = R_{e} /R_{e}^{0} ,\begin{array}{*{20}c} {} \\ \end{array} \zeta ^{\prime} = Eh/E_{{}}^{0} h^{0} \hfill \\ X^{\prime} = X/X^{0} ,P_{m}^{\prime } = P_{m} /P_{m}^{0} ,\begin{array}{*{20}c} {} \\ \end{array} K_{{}}^{\prime } = K_{{}} /K_{{}}^{0} \hfill \\ \end{gathered} \right.$$

**Figure 6:**
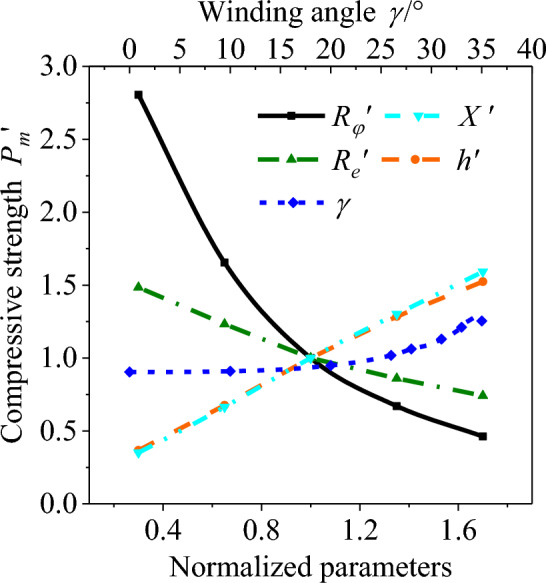
Influence curve of main design parameters on compressive strength.

**Figure 7:**
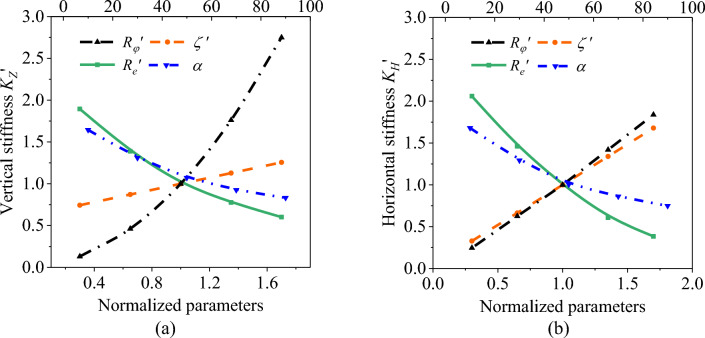
Influence curve of main design parameters on vertical and horizontal stiffness (**a**) Variation curve of vertical stiffness; (**b**) Variation curve of horizontal stiffness.

In Eq. (15), *P*_*m*_ is the compressive strength, which refers to the limit pressure causing the rupture of an AS under pressurization; *ζ* is the fiber structure parameter, which is a product of elastic modulus *E* and thickness of filament layer *h*; $R_{\varphi }^{0}$, $R_{e}^{0}$, *E*^0^, *h*^0^,* X*^0^, $P_{m}^{0}$ and *K*^0^ are the parameters of the 15 T AS; $R^{\prime}_{\varphi }$, $R^{\prime}_{e}$, $\zeta ^{\prime}$, $X^{\prime}$, $P_{m}^{\prime }$ and $K_{{}}^{\prime }$ are the normalized parameters. The compressive strength, vertical stiffness, and horizontal stiffness of the AS are calculated after altering the design parameters as presented in Figs. 6 and 7. The oriented angle has barely affected the strength of the AS, and the initial winding angle exerts little effect on the stiffness of the AS. Therefore, Figs. 6 and 7 do not demonstrate the variation of compressive strength with the oriented angle or the variation of stiffness with the initial winding angle. Additionally, the oriented angle is typically designed within the range of [0°, 90°], and the initial winding angle is usually within the range of [0°, 35°] due to the structural limitations of the AS guide seat and the requirements of the winding process in the bellows forming process^30^.

As revealed in Fig. 6, the strength of the AS decreases with the increase of effective radius and bellow radius, becomes larger with the increase of material strength and thickness of filament layer, goes up and then down with the increase of initial winding angle. The compressive strength varies with design parameters within their entire range of variation including bellow radius (234.3%), material strength (117.8%), thickness of filament layer (115.7%), effective radius (74.2%), and initial winding angle (36.9%). This reflects the influence of these design parameters on the strength of the AS. Among them, bellow radius has the strongest influence on the strength of the AS.

As shown in Fig. 7, the vertical and horizontal stiffness of the AS increases with the increase of effective radius and material structure parameters, and goes down with the increase of bellow radius and oriented angle. The vertical stiffness varies with design parameters within their entire range of variation including effective radius (261.8%), bellow radius (129.3%), oriented angle (81.2%), and thickness of filament layer and elastic modulus (51.25%). Meanwhile, the horizontal stiffness varies with design parameters within their entire range of variation including bellow radius (167.6%), effective radius (159.3%), thickness of filament layer and elastic modulus (135.1%), and oriented angle (93.0%). This reflects to what degree these design parameters affect the stiffness of the AS. Among them, effective radius exerts the strongest effect on the vertical stiffness, while bellow radius has the highest influence on the horizontal stiffness.

#### Optimization focus of design parameters

Effective radius is often certain for a specific model and size of AS. For this reason, it is not suitable for optimization among the design parameters of a specific prototype. After analyzing the dominating factors affecting the strength and stiffness of the AS, the influence of design parameters on strength and stiffness is detailed in Table 5. In the table, “ + ” indicates the positive correlation between parameters, while “−” stands for the negative correlation between parameters.

**Table 5:** Influence of design parameters on the mechanical properties of an AS.

Description		Compressive strength	Vertical stiffness	Horizontal stiffness
Geometrical structure parameters	Bellow radius *R*_*φ*_	− 234.3%	− 129.3%	− 167.58%
	Oriented angle *α*	− 6.4%	− 81.2%	− 93.0%
Material characteristics parameters	Material strength *X*	+ 117.8%	0%	0%
	Elastic modulus *E*	0%	+ 51.25%	+ 135.1%
	Thickness of filament layer *h*	115.7%		
Filament winding parameters	Initial winding angle *γ*	+ 36.9%	+ 4.9%	+ 11.2%

Therefore, optimization design focuses on improving the strength of an AS and lowering its stiffness. The design parameters are optimized in three categories as given in Table 6:

1. Design parameters affect strength or stiffness. For instance, material strength exerts an effect on strength, but elastic modulus affects stiffness only. In this case, the bellows may be made of a fiber composite with lower elastic modulus but higher material strength, so as to enhance the comprehensive performance (high strength and low stiffness) of the AS.
2. Design parameters affect both strength and stiffness, but their influence on strength or stiffness is slight and basically ignorable. For instance, the oriented angle affects among the geometrical structure parameters vertical and horizontal stiffness significantly (− 81.2% and − 93.0%), but has a little influence on compressive strength (− 6.4%). The initial winding angle exerts a significant effect on compressive strength (+ 36.9%), but has an insignificant impact on vertical and horizontal stiffness (+ 4.9% and + 11.2%). As a result, the oriented angle may be optimized for stiffness separately, while the initial winding angle is considered in the separate optimization design for strength. In this way, the comprehensive performance of the AS can be optimal.
3. Design parameters affect both strength and stiffness, and have a positive or negative correlation with them. For instance, the bellow radius among the geometrical structure parameters is negatively correlated with the compressive strength, vertical and horizontal stiffness of the AS (− 234.3%, − 129.3%, and − 167.58%). The thickness of filament layer among the material characteristics parameters has a positive correlation with the compressive strength, vertical and horizontal stiffness of the AS (115.7%, + 51.25%, and + 135.1%). In this case, if the bellow radius or thickness of filament layer is adjusted alone, it is impossible to meet the requirement for improved strength and lowered stiffness in optimization design. The optimization design intends to make the safety coefficient of an AS exceed 10 with as low stiffness as possible, so that the grid method is employed to optimize the bellow radius and thickness of filament layer. If the calculation error is 10%, the objective function and constraint of the optimization design is as follows:
  16 $$\left\{ \begin{gathered} \min K\left( {R_{e} ,h} \right) \hfill \\ s.t.\left\{ {P_{m} \ge 11.2 \cdot P_{e} } \right\} \hfill \\ \end{gathered} \right.$$

**Table 6:** Main design parameters of the AS before and after optimization.

15 T		15TG	
Item	Value	Item	Value
$R_{e}$	141 mm	$R_{e}$	141 mm
$R_{\varphi }$	22.5 mm	$R_{\varphi }$	15 mm
*β*	54°	*β*	90°
*h*	4.4 mm	*h*	2.2 mm
*γ*	28.9°	*γ*	30.1°

### Test verification of optimization design results

**Figure 8:**
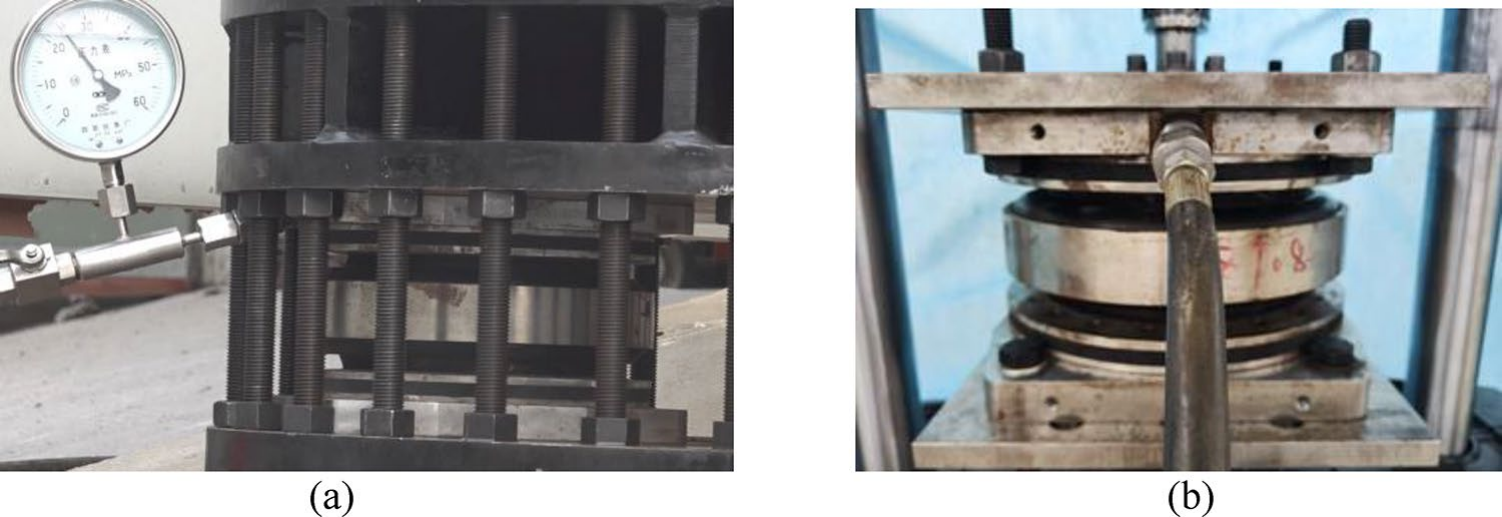
Tests of mechanical properties for the optimized prototype (**a**) Compressive strength test; (**b**) Stiffness test.

The optimization design was carried out with the parameters of the 15 T prototype. The rated working pressure *P*_*e*_ of the 15 T prototype was 2.3 Mpa. Considering that its horizontal stiffness was considerably greater than vertical stiffness, the minimum horizontal stiffness was the target of this optimization design. The prototype has been optimized using a dual-membrane low-stiffness structural solution, following the method outlined in "Integrated optimization design of structural parameters". The model of the optimized prototype is 15TG. The main design parameters of the AS before and after optimization are given in Table 6. The strength and stiffness of the 15TG AS were tested as shown in Fig. 8. The test results are summed up in Table 7.

**Table 7:** Calculated and test results of mechanical properties for the optimized prototype.

Description	15TG			15 T	Effectiveness of optimization design
	Calculated result	Test result	Calculation error	Test result	
*K*_*Z*_ (kN/mm)	4.24	4.02	5.5%	5.51	− 27.0%
*K*_*H*_ (kN/mm)	3.09	3.38	8.6%	13.80	− 75.5%
*P*_*m*_ (MPa)/safety coefficient	26.1/11.3	24.0/10.4	8.8%	27.0/11.7	Safety coefficient ≥ 10

As shown in Tables 6 and 7, the structure and design parameters are optimized when the overall size of the AS (i.e. effective radius) remains unchanged. While the safety coefficient of the AS is guaranteed to be above 10, the optimized prototype has its vertical stiffness lowered by 27.0%, and horizontal stiffness reduced by 75.5%. Evidently, the optimization design is productive, proving the correctness of the stiffness parameterization design model and the effectiveness of the optimization design method.

## Conclusions

In this paper, a mechanical model for the bellows is built on the basis of the shell theory. It is solved with the precise transfer matrix and boundary conditions to obtain the state vector of the bellows. The coupling relationship between vector and parameter is analyzed by iterative method. In this way, the vertical and horizontal stiffness of the AS is calculated and analyzed for design optimization.

1. A theoretical model is constructed to solve the stiffness of an AS for ships. A mechanical model for the bellows is built on the basis of the shell theory. Based on the precise transfer matrix and iteration methods, the parameterization method is proposed to calculate the stiffness of the bellows, and parameterize the vertical and horizontal stiffness of the AS. As revealed in the analysis, the bellow stiffness plays a significant role in the stiffness of the AS, and gradually dominates its overall stiffness, when the structural strength of the bellows is enhanced.
2. The low-stiffness optimization design is presented for an AS for ships. A dual-membrane low-stiffness structure is proposed to achieve the low-stiffness optimization design for the high-strength bellows. After analyzing the dominating factors affecting the strength and stiffness of the AS, the optimization design is determined with the focus on parameters as follows: (1) The fiber composite with high strength and low elastic modulus is selected to produce the bellows; (2) The oriented angle and initial winding angle exert a significant effect on the strength or stiffness separately, so that they may be addressed separately in the optimization design; (3) There is a strong coupling between the bellow radius and thickness of filament layer in terms of their influence on strength and stiffness, so that the grid method can be borrowed in the optimization design.
3. Three types of prototype and one optimized prototype are tested for performance. The test results reveal that the stiffness calculation error of such four prototypes is below 10%, proving the correctness of the parameterized design model for the stiffness of the AS. When the safety coefficient of the optimized prototype is guaranteed to be above 10, the vertical stiffness is lowered by 27.0% and the horizontal stiffness is reduced by 75.5%, which verifies the effectiveness of the stiffness optimization design method.

## Supplementary Information

Supplementary Information.

## Data Availability

The data used to support the findings of this study are available from the corresponding author upon request.
