# Supplementary material for: Stiffness analysis and structural optimization design of an air spring for ships
Source: Sci Rep. 2024 Jun 25;14:14650. doi: 10.1038/s41598-024-62581-3 (PMC11637151; doi:10.1038/s41598-024-62581-3)
Supplement: Supplementary file 1 — Supplementary Information. [file 41598_2024_62581_MOESM1_ESM.doc]

**Appendix:**

1. Elements of the conversion matrix ***B***(*ϕ*)

, , , , , ，, , ,

, , , , ,

*A_ij_* and *D_ij_* are the stiffness coefficients of composite ^[31]^.

1. Elements of the stiffness matrix ***C***(*ϕ*)

$$C_{8,8}=-2 \frac{R'_{0}}{R_{0}}$$
